# Conversion of Phosphogypsum into Porous Calcium Silicate Hydrate for the Removal and Recycling of Pb(II) and Cd(II) from Wastewater

**DOI:** 10.3390/molecules29112665

**Published:** 2024-06-04

**Authors:** Gangan Wang, Chaoyi Chen, Junqi Li, Yuanpei Lan, Xin Lin, Jiahang Chen

**Affiliations:** 1 School of Materials and Metallurgy, Guizhou University, Guiyang 550025, China; wgg0809@126.com (G.W.); yplan@gzu.edu.cn (Y.L.); 15111575229@163.com (X.L.); 18275207912@163.com (J.C.); 2 Guizhou Province Dual Carbon and New Energy Technology Innovation and Development Research Institute, Guiyang 550025, China

**Keywords:** phosphogypsum, porous calcium silicate hydrate, competitive interaction, adsorption mechanism, stepwise desorption

## Abstract

The discharge of lead and cadmium wastewater, along with the pollution caused by phosphogypsum, represents a particularly urgent environmental issue. This study employed a straightforward hydrothermal method to convert phosphogypsum into porous calcium silicate hydrate (P-CSH), which was then used to remove and recover Pb(II) and Cd(II) from wastewater. The adsorption capacities of P-CSH for Pb(II) and Cd(II) were notably high at 989.3 mg/g and 290.3 mg/g, respectively. The adsorption processes adhered to the pseudo-second-order kinetics model and the Langmuir isotherm model. Due to identical adsorption sites on P-CSH for both Pb(II) and Cd(II), competitive interaction occurred when both ions were present simultaneously. Additionally, the adsorption efficacy was minimally impacted by the presence of common coexisting cations in wastewater. The dominant mechanisms for removing Pb(II) and Cd(II) via P-CSH were chemical precipitation and surface complexation. Moreover, the adsorbed heavy metals were efficiently separated and reclaimed from the wastewater through a stepwise desorption process. The primary components of the residue from stepwise desorption were quartz and amorphous SiO_2_. Following dissolution via pressurized alkaline leaching, this residue could be recycled for synthesizing P-CSH. This research offered a new strategy for the resourceful use of phosphogypsum and heavy metal wastewater.

## 1. Introduction

The ecological safety issues arising from the extensive discharge of heavy metal wastewater constitute a significant global challenge [1,2,3]. Moreover, the complexity of this issue is heightened by the fact that heavy metal pollution generally results from the coexistence of multiple heavy metals, rather than a single contaminant. This composite pollution increases ecological risks and complicates remediation efforts significantly [4,5]. Lead (Pb(II)) often naturally co-occurs with cadmium (Cd(II)), both of which are extensively utilized heavy metals known for their considerable toxicity. Consequently, pollution involving both Pb(II) and Cd(II) is commonly reported [4,6,7]. Various methods have been deployed to extract heavy metals from wastewater, such as chemical precipitation [8], coagulation [9], electrochemical techniques [10], membrane filtration [11], and adsorption [12]. Of these, adsorption is particularly noteworthy due to its cost-effectiveness, low-energy-requirement, high removal efficiency, and capability to recover heavy metals from wastewater [13,14]. To enhance the potential of the adsorption method in heavy metal wastewater treatment, it is crucial to continue developing adsorbents that are cost-effective, high performance, simple to prepare, and environmentally friendly.

To remove Pb(II) and Cd(II) from wastewater, various adsorbents have been developed, including modified biochar, metal–organic frameworks, aerogels, and silicate inorganic materials [15,16,17,18,19]. Among these, calcium silicate hydrate (C-S-H)—a type of silicate inorganic material—possesses a highly developed porous structure, a large specific surface area, the ability to self-release OH^−^, and is non-toxic and simple to synthesize. It is extensively applied for the removal of heavy metal ions from wastewater [20,21]. To further reduce the production costs of C-S-H, the use of industrial wastes as raw materials for synthesizing C-S-H is increasingly attracting attention [22,23].

Phosphogypsum, the principal solid waste from wet phosphoric acid manufacturing, is mainly composed of calcium sulfate dihydrate. It also contains trace amounts of impurities, including phosphorus, fluorine, silicon, and organic materials [24]. The production process yields about 4 to 5 tons of phosphogypsum for each ton of phosphoric acid produced [25]. Annually, approximately 280 million tons of phosphogypsum are produced worldwide, but only about 15% is utilized in creating building materials, soil amendments, and the production of calcium carbonate and ammonium sulfate. The vast majority is dealt with through surface storage or ocean dumping, leading to significant ecological damage and wastefulness of resources [25,26,27]. If phosphogypsum could be converted into C-S-H adsorbents for treating Pb(II) and Cd(II) in wastewater, it would both broaden the means of utilization for this byproduct and significantly reduce the costs associated with producing C-S-H.

In this study, a straightforward hydrothermal method was employed to convert phosphogypsum into P-CSH, which was then used for the removal of Pb(II) and Cd(II) from wastewater. Subsequently, the adsorbed contaminants were effectively recovered and separated through a stepwise desorption process. This research comprehensively explored the adsorption kinetics, isotherms, and the impact of both competitive or synergistic adsorption, as well as the presence of coexisting ions on the performance of P-CSH in capturing Pb(II) and Cd(II). Detailed analyses utilizing XRD, SEM-EDS, FT-IR, and ^29^Si MAS NMR characterization methods elucidated the adsorption mechanisms. By converting phosphogypsum into a high-performance adsorbent, this study achieved the dual objectives of “waste treating waste” and “turning waste into treasure”, offering innovative strategies for the resourceful use of phosphogypsum and the remediation of heavy metal-contaminated wastewater.

## 2. Results and Discussion

### 2.1. Characterizations of Phosphogypsum and P-CSH

**Figure 1:**
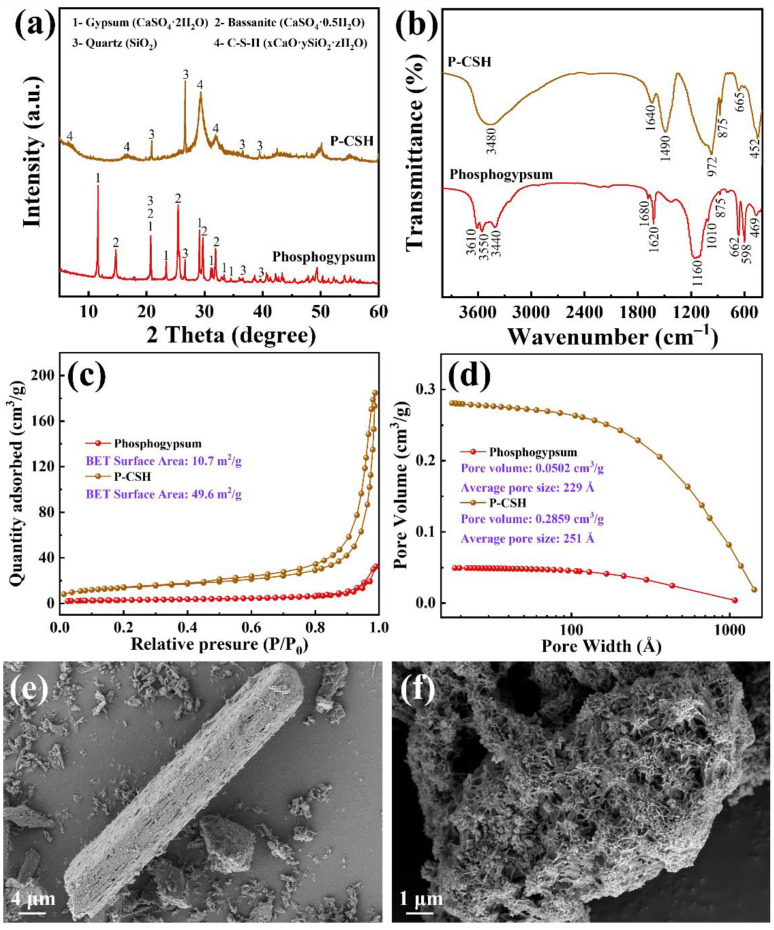
(**a**) XRD pattern, (**b**) FT-IR spectra, (**c**) N_2_ adsorption–desorption isotherms, (**d**) pore diameter distribution, and (**e**,**f**) SEM images of phosphogypsum and P-CSH, respectively.

Figure 1a shows the XRD patterns of phosphogypsum and P-CSH. The predominant phases identified in phosphogypsum are gypsum (CaSO_4_·2H_2_O, JCPDS No. 01-074-1433), bassanite (CaSO_4_·0.5H_2_O, JCPDS No. 00-033-0310), and quartz (SiO_2_, JCPDS No. 01-079-1906). Following the conversion process, the characteristic peaks of gypsum and bassanite are no longer observed, while the peak of quartz intensifies significantly. Additionally, characteristic peaks of C-S-H (xCaO·ySiO_2_·zH_2_O, JCPDS No. 00-006-0013) are detected at 7.68°, 16.37°, 29.16°, and 31.94°, indicating the successful conversion of gypsum and bassanite in phosphogypsum into C-S-H.

Figure 1b presents the FT-IR spectra of phosphogypsum and P-CSH. Following the conversion process, the asymmetric bending vibrations of [SO_4_^2−^] at 598 cm^−1^ and 662 cm^−1^, along with the asymmetric stretching vibrations of [SO_4_^2−^] at 1010 cm^−1^ and 1160 cm^−1^, are no longer detected [28,29], signaling the substantial removal of [SO_4_^2−^] from phosphogypsum. In both materials, the bending vibrations of [OH^−^] are identified between 3440 cm^−1^ and 3610 cm^−1^, and the vibrational peaks of water molecules appear between 1620 cm^−1^ and 1640 cm^−1^ [30,31]. These observations indicate significant quantities of free and bound water in both samples, aligning with the XRD results (Figure 1a). Furthermore, a characteristic vibration peak of [PO_4_^3−^] at 875 cm^−1^ is noted in both phosphogypsum and P-CSH [32,33], reflecting the presence of minor phosphate contents. In P-CSH, detections include the asymmetric stretch of Si-O-Si at 972 cm^−1^, bending of Si-O-Si at 665 cm^−1^, and internal deformation of Si-O tetrahedra at 451 cm^−1^ [23,34,35], demonstrating the presence of Si-O tetrahedral structures. Moreover, a distinctive peak of Ca-O at 1490 cm^−1^ indicates the synthesized P-CSH’s capability to release Ca^2+^ and OH^−^ in aqueous solutions [23].

Figure 1c, d depicts the N_2_ adsorption–desorption isotherms and pore diameter distribution for phosphogypsum and P-CSH, respectively. Initially, phosphogypsum exhibits a specific surface area of only 10.7 m^2^/g and a total pore volume of 0.0502 cm^3^/g. Post-conversion, these values for P-CSH significantly increase to 49.6 m^2^/g for the specific surface area and 0.2859 cm^3^/g for the pore volume, enhancing the availability of adsorption sites essential for the removal of Pb(II) and Cd(II) [34]. Furthermore, P-CSH primarily features a mesoporous structure with an average pore size of 229 Å. This structural attribute not only aids mass transfer during adsorption, but also contributes to increased stability of the material [36,37]. The N_2_ adsorption–desorption isotherm (Figure 1c) for P-CSH is classified as type IV, featuring a type-H3 hysteresis loop in the latter stages and lacking a distinct saturation plateau, suggesting an irregular porous structure [38]. The SEM image presented in Figure 1f validates the findings of the analysis.

### 2.2. Adsorption Performance of Pb(II) and Cd(II)

#### 2.2.1. Effect of Initial pH

**Figure 2:**
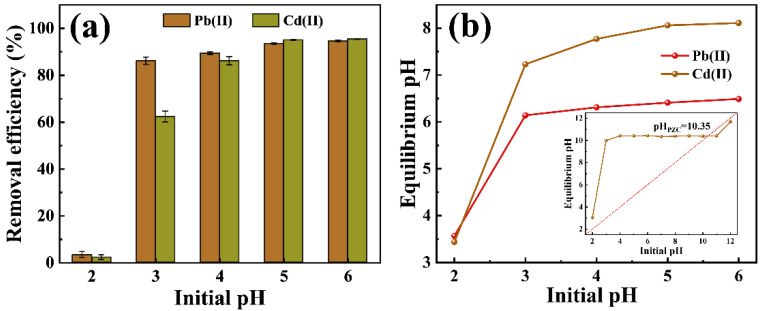
(**a**) Effect of initial pH on the removal efficacy of Pb(II) and Cd(II); (**b**) the relationship between initial pH and equilibrium pH, and the pH_PZC_ of P-CSH.

Figure 2a illustrates the effect of initial pH on the removal efficiency of Pb(II) and Cd(II). At an initial pH of 2.0, the removal efficiencies achieved by P-CSH for Pb(II) and Cd(II) were notably low, at 3.23% and 2.17% respectively. The equilibrium pH values of the solutions post-adsorption were 3.57 and 3.44, respectively, as demonstrated in Figure 2b. With an increase in the initial pH, there was a rapid rise in the equilibrium pH of the solutions, accompanied by a gradual improvement in the removal efficiencies for both Pb(II) and Cd(II). At an initial pH of 5.0, the removal efficiencies of P-CSH for Pb(II) and Cd(II) reached stability. Further increases in initial pH did not significantly affect these removal efficiencies. Consequently, all subsequent experiments were performed at an initial pH of 5.0.

The point of zero charge (pH_PZC_) of P-CSH was determined to be 10.35 using the pH drift method [39,40]. Considering the relationship between the initial and equilibrium pH values during the adsorption of Pb(II) and Cd(II) by P-CSH (Figure 2b), the pH of the solution during adsorption always remained below pH_PZC_. This indicates that the surface of P-CSH was positively charged during the adsorption process. As a result, there was electrostatic repulsion between the positively charged surface of P-CSH and the Pb(II) and Cd(II) in the solution, suggesting that electrostatic adsorption did not occur during the process [34].

#### 2.2.2. Adsorption Kinetics

**Figure 3:**
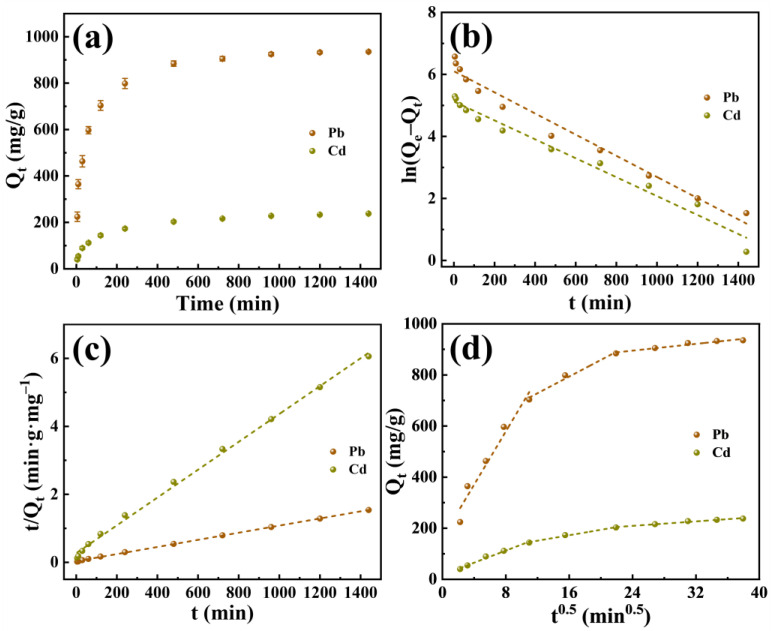
(**a**) The relationship between Q_t_ of P-CSH for Pb(II) and Cd(II) and the duration of adsorption; (**b**) pseudo-first-order kinetics; (**c**) pseudo-second-order kinetics; (**d**) intra-particle diffusion kinetics.

Figure 3a illustrates the relationship between the adsorption capacity (Q_t_) of P-CSH for Pb(II) and Cd(II) and the duration of adsorption. As the time is extended, the Q_t_ of P-CSH to adsorb Pb(II) and Cd(II) progressively increases. Specifically, at 720 min, the Q_t_ is 905.1 mg/g for Pb(II) and 216.0 mg/g for Cd(II). On further extending the time, the increase in Q_t_ occurs at a slower rate; at 1440 min, Q_t_ reach 935.4 mg/g for Pb(II) and 237.7 mg/g for Cd(II).

The adsorption scatter plots were modeled using pseudo-first-order, pseudo-second-order, and intra-particle diffusion kinetics, as delineated in Equations (1) to (3), respectively [21,41]. The results of these fittings are displayed in Figure 3b–d and Table 1.

(1) $$ln(Q_{e}-Q_{t})=ln(Q_{e})-k_{1}t$$

(2) $$\frac{t}{Q_{t}}=\frac{1}{k_{2}Q_{e}^{2}}+\frac{t}{Q_{e}}$$

(3) $$Q_{t}=k_{i}t^{0.5}+C$$

where Q_e_ (mg/g) represents the adsorption capacity at equilibrium; Q_t_ is the adsorption capacity at time t; t (min) is the adsorption time; k_1_, k_2_, and k_i_ are the rate constants for pseudo-first-order, pseudo-second-order, and intra-particle diffusion kinetics, respectively; and C (mg/g) is the intercept.

Table 1 reveals that the pseudo-second-order kinetics provide a more accurate depiction of the adsorption behavior of P-CSH toward Pb(II) and Cd(II). According to this model, the calculated Q_e_ values for P-CSH with Pb(II) and Cd(II) are 954.2 mg/g and 244.1 mg/g, respectively. These values closely align with the experimental results. This congruence suggests that the adsorption of Pb(II) and Cd(II) by P-CSH adheres to the pseudo-second-order kinetics, predominantly governed by chemical reactions [42,43].

The intra-particle diffusion model was employed to further investigate the rate-controlling steps within the adsorption process. Generally, this process encompasses three primary stages: (a) the diffusion of pollutants to the external surface of the adsorbent, (b) from the external surface through the boundary layer to the pores of the adsorbent, and (c) subsequently diffusing to the internal surface [44]. For P-CSH when adsorbing Pb(II) and Cd(II), the process distinctly divides into three phases, with the intra-particle diffusion rate constants demonstrating k_i1_ > k_i2_ > k_i3_ (Figure 3d and Table 1). This implies that, during the initial adsorption stage, Pb(II) and Cd(II) swiftly diffuse to the external surface of P-CSH, then progressively toward the pores and the internal surface, with the rate of adsorption gradually diminishing until equilibrium is achieved. The fitting curve of the intra-particle diffusion model does not intersect the origin, indicating that intra-particle diffusion is not the sole rate-limiting step in the adsorption of Pb(II) and Cd(II) by P-CSH [44,45]. This finding aligns with the results analyzed under pseudo-second-order kinetics.

#### 2.2.3. Adsorption Isotherms

**Figure 4:**
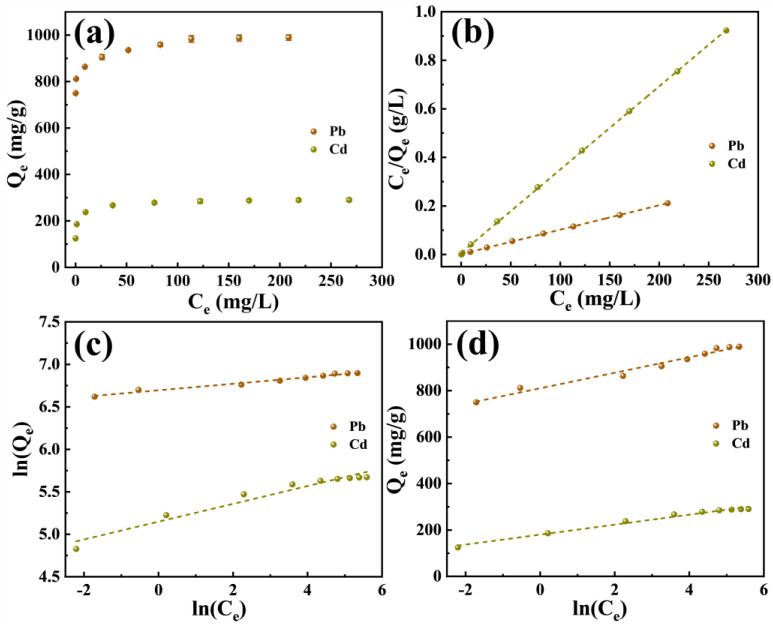
(**a**) The relationship between the Q_e_ of P-CSH for Pb(II) and Cd(II) and C_e_; (**b**) Langmuir; (**c**) Freundlich; and (**d**) Temkin isotherms.

The association between P-CSH’s equilibrium adsorption capacities (Q_e_) for Pb(II) and Cd(II) and their respective equilibrium concentrations (C_e_) is depicted in Figure 4a. As these concentrations increase, there is a corresponding rise in P-CSH’s Q_e_. Specifically, at C_e_ of 208.6 mg/L for Pb(II) and 267.8 mg/L for Cd(II), Q_e_ values were found to be 989.3 mg/g and 290.3 mg/g, respectively. These data were analyzed using the Langmuir, Freundlich, and Temkin adsorption isotherm models, as represented in Equations (4) to (6), respectively [44,46]. The results of these fits are presented in Figure 4b–d and in Table 2.

(4) $$\frac{C_{e}}{Q_{e}}=\frac{C_{e}}{Q_{m}}+\frac{1}{K_{L}Q_{m}}$$

(5) $$ln(Q_{e})=ln(K_{F})+\frac{1}{n}ln(C_{e})$$

(6) $$Q_{e}=\frac{RT}{b}ln(K_{T})+\frac{RT}{b}ln(C_{e})$$

where Q_e_ (mg/g) represents the equilibrium adsorption capacity; Q_m_ (mg/g) represents the maximum adsorption capacity; C_e_ (mg/L) represents the equilibrium concentration; K_L_ (L/mg), K_F_ (mg^(1−1/n)^·L^1/n^·g^−1^), and K_T_ (L/mg) are the Langmuir, Freundlich, and Temkin adsorption isotherm constants, respectively; n is the Freundlich index; R is the gas constant; T (K) is the absolute temperature; and b represents the heat of adsorption.

The Langmuir isotherm model more precisely characterizes the adsorption behavior of P-CSH toward Pb(II) and Cd(II), with a correlation coefficient (R^2^) exceeding 0.9990. This high correlation suggests that the adsorption of both Pb(II) and Cd(II) by P-CSH involves monolayer adsorption [47]. According to the Langmuir isotherm model, the saturated adsorption capacities of P-CSH for Pb(II) and Cd(II) are 993.1 mg/g and 291.5 mg/g, respectively, aligning well with the experimental values.

The essential characteristic of the Langmuir isotherm model can be represented by the dimensionless separation factor (R_L_, Equation (7)) [42]. An R_L_ value of 0 indicates an irreversible adsorption process; a value between 0 and 1 suggests that adsorption occurs readily; R_L_ equal to 1 signifies a linear adsorption process; and an R_L_ greater than 1 indicates that the adsorption process is difficult to occur.

(7) $$R_{L}=\frac{1}{{1+K}_{L}C_{0}}$$

where R_L_ is the separation factor; K_L_ is the Langmuir isotherm constant; and C_0_ (mg/L) refers to the initial concentration.

**Figure 5:**
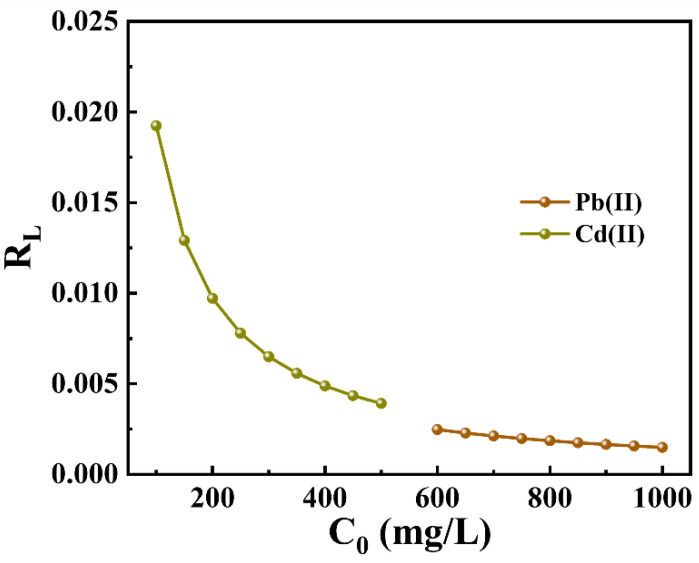
Separation factors (R_L_) of Pb(II) and Cd(II) by P-CSH.

The R_L_ values for Pb(II) and Cd(II) are presented in Figure 5. The R_L_ values for the adsorption process of Pb(II) and Cd(II) by P-CSH vary from 0 to 1.0 and exhibit a gradual decrease as the initial concentration increases. This trend suggests that P-CSH possesses strong adsorption capabilities for Pb(II) and Cd(II). Furthermore, an increase in the initial concentration facilitates the adsorption of these heavy metals by P-CSH.

#### 2.2.4. Competitive or Cooperative Adsorption

**Figure 6:**
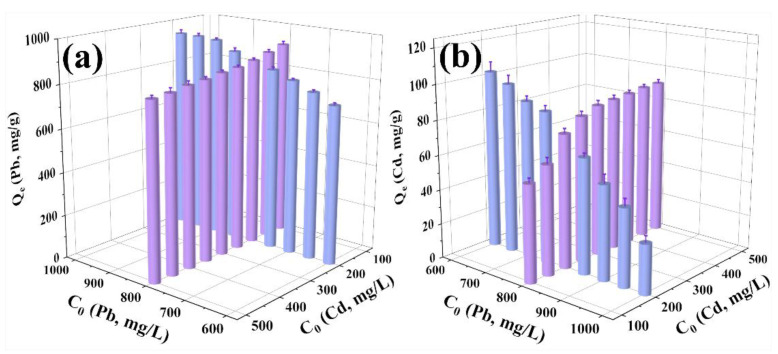
The Q_e_ of P-CSH for (**a**) Pb(II) and (**b**) Cd(II) at different initial concentrations of Pb(II) and Cd(II).

To further investigate the adsorption behavior of P-CSH in the presence of both Pb(II) and Cd(II), we examined how varying the initial concentrations of Pb(II) and Cd(II) affects the adsorption capacities of P-CSH. The results are presented in Figure 6.

From Figure 6, it can be observed that, under a constant initial Cd(II) concentration of 200 mg/L, the Q_e_ of P-CSH for Pb(II) increases with rising initial Pb(II) concentrations. Conversely, when the initial Pb(II) concentration is held at 800 mg/L, the Q_e_ of P-CSH for Pb(II) declines as the initial Cd(II) concentration increases (Figure 6a). Similarly, with Pb(II) maintained at 800 mg/L, the Q_e_ of P-CSH for Cd(II) enhances with increasing initial concentrations of Cd(II). However, when the initial Cd(II) concentration is fixed at 200 mg/L, the Q_e_ of P-CSH for Cd(II) diminishes as the initial Pb(II) concentration increases (Figure 6b).

Equation (8) represents the formula for calculating the interaction factor (P). When P exceeds 1.0, it indicates a synergistic effect between Pb(II) and Cd(II). A *p*-value of 0 signifies that there is no interaction between Pb(II) and Cd(II), while a *p*-value less than 1.0 signals a competitive interaction between the two metals [4,48].

(8) $$P=Q_{max,binary}/Q_{max,single}$$

where P represents the interaction factor; Q_max, binary_ (mg/g) represents the maximum adsorption capacity when both Pb(II) and Cd(II) are adsorbed simultaneously; and Q_max, single_ (mg/g) refers to the maximum adsorption capacity when either Pb(II) or Cd(II) is adsorbed individually.

**Figure 7:**
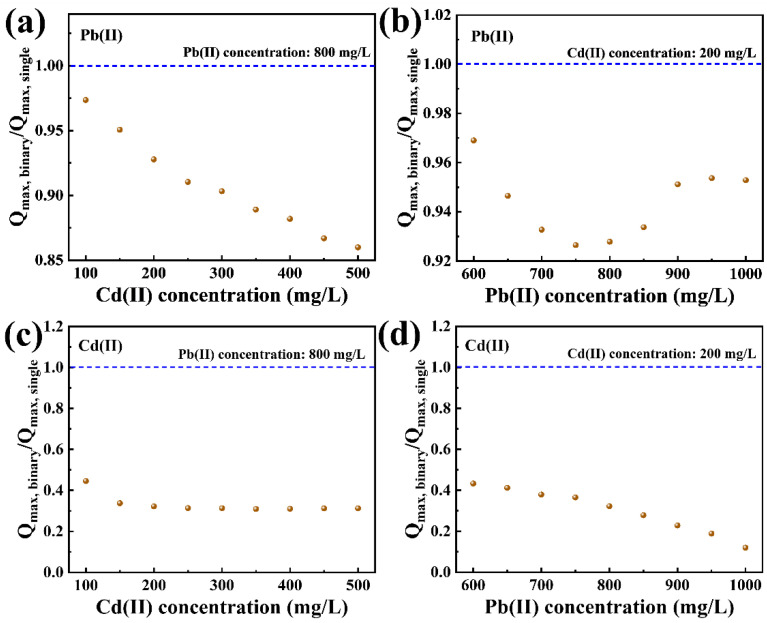
The interaction factors of Pb(II) at varied initial concentrations of (**a**) Cd(II) and (**b**) Pb(II); and the interaction factors of Cd(II) at varied initial concentrations of (**c**) Cd(II) and (**d**) Pb(II).

Figure 7 illustrates that, under various initial concentrations of Pb(II) and Cd(II), the interaction factors for both metals are less than 1.0. This indicates that, when Pb(II) and Cd(II) are simultaneously adsorbed by P-CSH, there is exclusively competitive interaction between them. This finding aligns with the experimental results presented in Figure 6.

#### 2.2.5. Effect of Coexisting Ions

**Figure 8:**
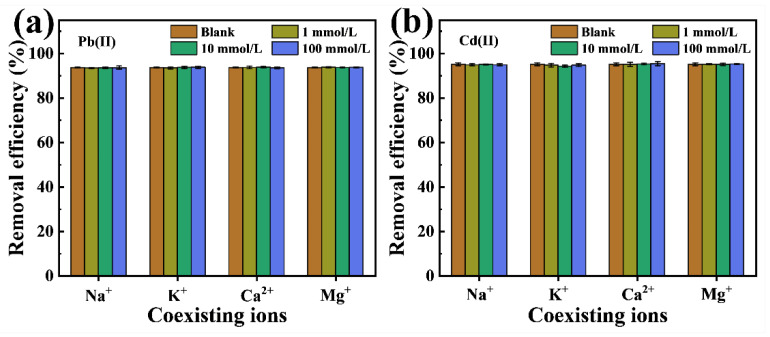
The effect of coexisting ions on the removal efficiency for (**a**) Pb(II) and (**b**) Cd(II), respectively.

In practical applications, adsorbents can be hindered by various coexisting ions in wastewater, affecting their capacity to adsorb Pb(II) and Cd(II). Typical cations in heavy metal wastewater, such as Na^+^, K^+^, Ca^2+^, and Mg^2+^, may contribute to this interference. To evaluate the efficacy of P-CSH in treating actual wastewater samples, a study was conducted on the impact of these common cations on the removal efficiencies of Pb(II) and Cd(II). The results are presented in Figure 8.

Generally, common coexisting cations in wastewater, such as Na^+^, K^+^, Ca^2+^, and Mg^2+^, typically compete for active sites on the surface of adsorbents via electrostatic adsorption [21,49], which can diminish the adsorption capacities of P-CSH for Pb(II) and Cd(II). However, with the pH at the pH_PZC_ of P-CSH being 10.35, its surface remains positively charged during the adsorption process (Figure 2b). This results in electrostatic repulsion with the coexisting cations in the solution. Consequently, these commonly occurring cations in wastewater have a negligible impact on the adsorption efficacy of P-CSH for Pb(II) and Cd(II), as illustrated in Figure 7. This suggests that P-CSH, converted from phosphogypsum, demonstrates robust resistance to cationic interference, making it well-suited for challenging real-world wastewater conditions.

### 2.3. Adsorption Mechanism

**Figure 9:**
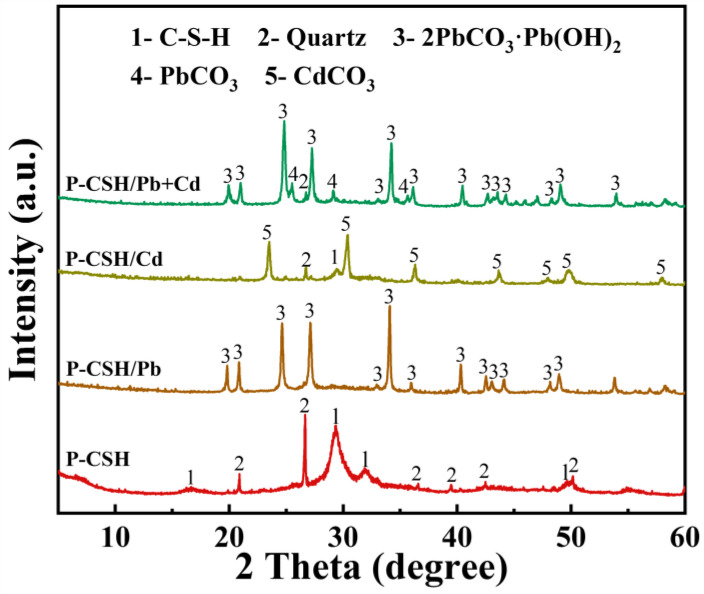
XRD patterns of P-CSH and adsorption products following both individual and simultaneous adsorptions of Pb(II) and Cd(II).

Figure 9 presents the XRD patterns of P-CSH and adsorption products formed when P-CSH adsorbs Pb(II) and Cd(II) individually and simultaneously. The products of individual Pb(II) and Cd(II) adsorption were predominantly hydrocerussite (2PbCO_3_·Pb(OH)_2_, JCPDS No. 00-010-0401) and otavite (CdCO_3_, JCPDS No. 00-042-1342), respectively. In contrast, the simultaneous adsorption of both Pb(II) and Cd(II) primarily produced hydrocerussite and cerussite (PbCO_3_, JCPDS No. 01-070-02052). Given that P-CSH demonstrated a significantly greater adsorption capacity for Pb(II) compared to Cd(II) at initial concentrations of 800 mg/L for Pb(II) and 200 mg/L for Cd(II) (Figure 6), no diffraction peaks associated with Cd(II) were observed in its adsorption product. Additionally, the presence of [CO_3_^2−^] in these products could be attributed to CO_2_ absorption from water or air during the adsorption or drying processes [50,51]. It was also observed that no diffraction peaks associated with C-S-H were detected in the products of Pb(II) adsorption alone or when both Pb(II) and Cd(II) were adsorbed simultaneously. Moreover, the diffraction peaks for C-S-H were notably weak in the products of Cd(II) adsorption alone, suggesting the substantial decomposition of C-S-H during the adsorption process.

**Figure 10:**
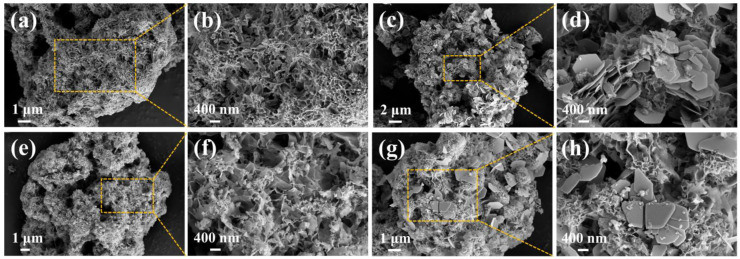
SEM images of P-CSH and adsorption products following both individual and simultaneous adsorptions of Pb(II) and Cd(II): (**a**,**b**) P-CSH; (**c**,**d**) P-CSH/Pb; (**e**,**f**) P-CSH/Cd; (**g**,**h**) P-CSH/Pb+Cd.

Figure 10 presents SEM images of P-CSH and adsorption products formed when P-CSH adsorbs Pb(II) and Cd(II) individually, as well as simultaneously. These images clearly demonstrate that the adsorption products extensively deposit on the surface of P-CSH, while the material maintains its porous structure. This observation suggests that P-CSH preserves its structural integrity during the adsorption process, which not only promotes the continuous release of Ca^2+^ and OH^−^ ions, but also generates additional binding sites that facilitate the removal of Pb(II) and Cd(II). When P-CSH adsorbs Pb(II) alone, the adsorption products predominantly exhibit a hexagonal plate-like structure (Figure 10c,d). According to the XRD analysis results depicted in Figure 9, these structures are primarily composed of hydrocerussite. For Cd(II) adsorption, the dominant morphologies observed are fine, curled flakes and dense, solid spherical forms (Figure 10e,f). When P-CSH adsorbs both Pb(II) and Cd(II) simultaneously, the morphology of the adsorption products becomes significantly more complex, displaying a mixture of irregular plate-like, solid spherical and fine, curled flake shapes, without a discernible pattern in their distribution (Figure 10g,h).

**Figure 11:**
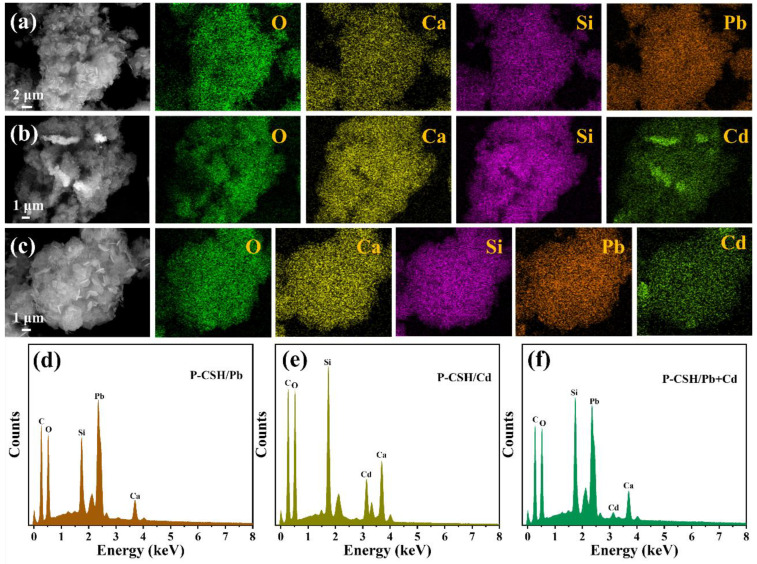
Elemental distribution maps and energy spectrum analysis of the adsorption products following both individual and simultaneous adsorptions of Pb(II) and Cd(II): (**a**,**d**) P-CSH/Pb; (**b**,**e**) P-CSH/Cd; (**c**,**f**) P-CSH/Pb+Cd.

Figure 11 presents the elemental distribution maps and energy spectrum analysis of the adsorption products when P-CSH adsorbs Pb(II) and Cd(II) either singly or simultaneously. The consistent element distribution across all three conditions implies that P-CSH shares common adsorption sites for Pb(II) and Cd(II). This shared site mechanism is likely responsible for the observed competitive adsorption between Pb(II) and Cd(II), depicted in Figure 6 and Figure 7. Additionally, residual traces of calcium, visible in Figure 11d–f, suggest that some C-S-H has remained intact. This is corroborated by the XRD analysis in Figure 9, indicating that these unreacted portions of C-S-H still possess the capacity to adsorb Pb(II) and Cd(II).

**Figure 12:**
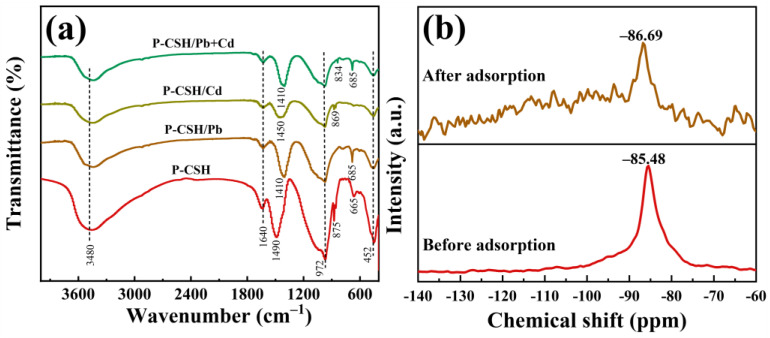
(**a**) FT-IR spectra of P-CSH and adsorption products following both individual and simultaneous adsorptions of Pb(II) and Cd(II); (**b**) ^29^Si MAS NMR spectra of P-CSH before and after adsorption.

Figure 12a displays the FT-IR spectra of P-CSH and adsorption products formed when P-CSH adsorbs Pb(II) and Cd(II) individually, as well as simultaneously. The spectra identify asymmetric stretching vibrations of Si-O-Si and internal deformation of Si-O tetrahedral structures at 972 cm^−1^ and 452 cm^−1^, respectively [23,34,35]. These spectral features confirm the presence of Si-O tetrahedral structures in the adsorption products, aligning with the analytical results for P-CSH shown in Figure 1b. Additionally, bending vibrations of [OH^−^] in water molecules and vibrations of water molecules themselves are detected at 3480 cm^−1^ and 1640 cm^−1^ [30,31], indicating significant amounts of free and bound water in the adsorption products. Characteristic peaks of hydrocerussite at 1410 cm^−1^ and 685 cm^−1^ are observed in P-CSH/Pb and P-CSH/Pb+Cd samples, while peaks for otavite are found at 869 cm^−1^ and 834 cm^−1^ [52,53], corroborating with the XRD findings presented in Figure 9.

Figure 12b displays the ^29^Si MAS NMR spectra of P-CSH before and after adsorption, illustrating notable changes in the chemical environment of Si within the material [35,36,54]. Before adsorption, the Si exhibits a predominant chemical shift at −85.48 ppm, suggesting its configuration predominantly in the form of Q_p_^2^ bridging Si-O tetrahedra. Following adsorption, the principal chemical shift of Si moves to −86.69 ppm, accompanied by the emergence of several weaker shifts ranging between −140 and −100 ppm. This indicates that post-adsorption, the Si within the adsorbent further polymerizes into more complex Q^4^ structures while retaining some Q_p_^2^ structures. These spectral changes corroborate the findings from XRD and energy spectrum analyses, detailed in Figure 9 and Figure 11.

In summary, we propose the potential adsorption mechanisms of P-CSH on Pb(II) and Cd(II):

(a) C-S-H spontaneously reacts with H^+^ and H_2_O present in the solution, consequently releasing Ca^2+^ and OH^−^ into the solution (Equations (9) and (10)) [22]:

[≡(SiO)_2_Ca](s) + 2H^+^(aq) = 2[≡Si-OH](s) + Ca^2+^(aq) (9)

[≡(SiO)_2_Ca](s) + 2H_2_O(I) = 2[≡Si-OH](s) + Ca^2+^(aq) + 2OH^−^(aq) (10)

(b) The structure of C-S-H mimics that of tobermorite, wherein Ca^2+^ are predominantly situated within the interlayers akin to the tobermorite structure, ensuring charge balance. Upon the substantial release of Ca^2+^ by C-S-H, the initial stable structure becomes destabilized. This destabilization prompts further polymerization of the initial bridging Si-O tetrahedra (Q_p_^2^), resulting in their transformation into a Q^4^ networked structure that reinforces the stability of the adsorbent (Equation (11)) [22].

[≡Si-OH](s) + [OH-Si≡](s) = [≡Si-O-Si≡](s) + H_2_O(I) (11)

(c) The OH^−^ released from the decomposition of C-S-H combines with Pb(II) and Cd(II) in the solution to form precipitates (Equations (12) and (13)), thereby facilitating the removal of Pb(II) and Cd(II) from wastewater.

Pb^2+^(aq) + 2OH^−^(aq) = Pb(OH)_2_(s) (12)

Cd^2+^(aq) + 2OH^−^(aq) = Cd(OH)_2_(s) (13)

Despite the similar solubility product constants (K_sp_) of Pb(OH)_2_ and Cd(OH)_2_, as well as the comparable ionic radii of Pb^2+^ and Cd^2+^ [21,55], P-CSH has demonstrated significantly different adsorption capacities for these ions. This phenomenon can be attributed to the differences in the hydration enthalpies of the ions. Specifically, the hydration enthalpies of Pb^2+^, Cd^2+^, and Ca^2+^ are −1481, −1807, and −1577 kJ/mol, respectively. These values indicate that Pb^2+^ is less stable in aqueous solutions compared to Cd^2+^. Moreover, during the adsorption process, P-CSH releases a substantial amount of Ca^2+^ into the solution, significantly enhancing its adsorption performance for Pb^2+^ over Cd^2+^. Additionally, during the adsorption and drying processes, the adsorption products undergo further transformation into minerals such as hydrocerussite, cerussite, and otavite, as illustrated in Figure 9.

(d) During the decomposition of C-S-H, numerous [≡Si-OH] groups form on the adsorbent surface. These groups facilitate the removal of Pb(II) and Cd(II) from wastewater via surface complexation (Equations (14) and (15)).

2[≡Si-OH] + Pb^2+^(aq) = [≡Si-O]_2_Pb + 2H^+^(aq) (14)

2[≡Si-OH] + Cd^2+^(aq) = [≡Si-O]_2_Cd + 2H^+^(aq) (15)

### 2.4. Stepwise Desorption and Recovery of Pb(II) and Cd(II)

Selective leaching with NaOH solution recovers 99.61% of Pb(II) from P-CSH/Pb+Cd, whereas the leaching efficiency for Cd(II) remains as low as 0.23%. Following alkaline leaching, the residue is subjected to a further leaching process using EDTA solution, which effectively extracts nearly all of Pb(II), Cd(II), and Ca present. These results indicate that a stepwise desorption protocol utilizing NaOH and EDTA solutions can efficiently recover and separate Pb(II) and Cd(II) from the adsorbed products.

**Figure 13:**
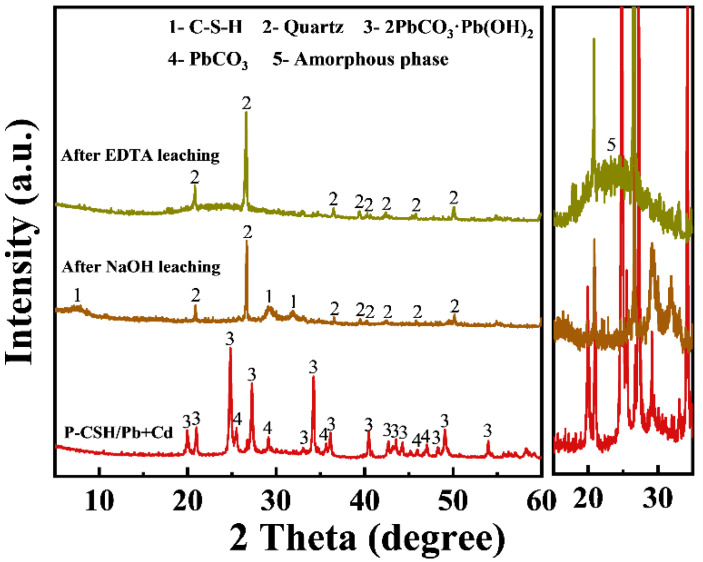
XRD patterns of P-CSH/Pb+Cd and the residues following stepwise desorption.

Figure 13 displays the XRD patterns of P-CSH/Pb+Cd and the residues following stepwise desorption. The diffraction peaks of hydrocerussite and cerussite in the solid samples are no longer visible after NaOH solution leaching, indicating that these minerals dissolved in the alkali solution, thus facilitating efficient Pb(II) extraction. Subsequent leaching of the residue with EDTA solution results in the complete disappearance of the C-S-H diffraction peaks in the solid samples, and a broad “dome-shaped peak” emerges between 15° and 35°, indicative of the presence of amorphous SiO_2_ in the EDTA-treated residues [56]. This solid residue, enriched in amorphous SiO_2_ and quartz, can be redissolved under alkaline pressurized conditions for reuse in the production of P-CSH [57,58].

**Figure 14:**
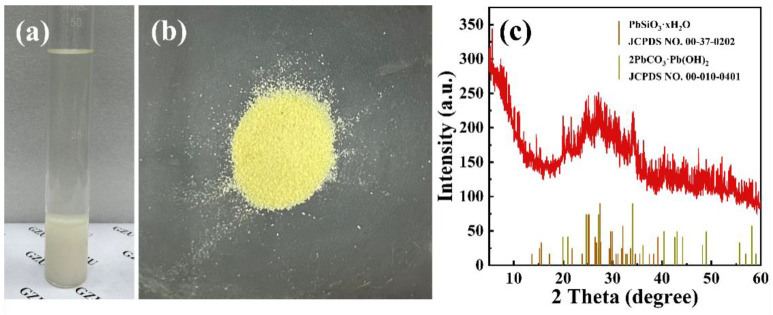
(**a**) Precipitation occurred after the Pb(II) desorption alkaline solution was left to stand at room temperature; (**b**) solid sample of the dried precipitate; (**c**) XRD pattern of the precipitate.

Figure 14a illustrates the precipitate that forms when the Pb(II) desorption alkali solution is left to stand at room temperature for 6 h; this precipitate, upon drying, appears as a beige powder, as depicted in Figure 14b. Figure 14c reveals that this precipitate, formed after settling, exhibits poor crystallinity. Its primary mineral phases are identified as hydrated lead silicate (PbSiO_3_·xH_2_O, JCPDS No. 00-037-0202) and hydrocerussite.

## 3. Materials and Methods

### 3.1. Materials

Phosphogypsum was sourced from a storage site in Guizhou Province, China. Prior to experimentation, it underwent a cleaning process involving deionized water to eliminate surface impurities, and was then air-dried at 105 °C for 24 h. The chemical composition of phosphogypsum was determined after digestion with inorganic acid, utilizing Inductively Coupled Plasma Optical Emission Spectrometry (ICP-OES, Agilent 720ES, Santa Clara, CA, USA). The primary constituents found were Ca and S, which comprised 21.61 wt.% and 17.27 wt.%, respectively. Additionally, minor components included 2.64 wt.% Si, 0.41 wt.% Al, and 0.35 wt.% Fe, among other elements. Chemical reagents, including Sodium Metasilicate Nonahydrate (Na_2_SiO_3_·9H_2_O, AR), Sodium Hydroxide (NaOH, AR), and Sodium Nitrate (NaNO_3_, AR), were procured from Aladdin Biochemical Technology Co., Ltd., Shanghai, China. Lead Nitrate (Pb(NO_3_)_2_, GR) and Cadmium Nitrate Tetrahydrate (Cd(NO_3_)_2_·4H_2_O, GR) were purchased from Sinopharm Chemical Reagent Co., Ltd., Shanghai, China. Nitric Acid (HNO_3_, AR) was purchased from Chuandong Chemical Co., Ltd., Chongqing, China. The deionized water used in the laboratory, with a resistivity of 18.25 MΩ·cm at 25 °C, was self-produced.

### 3.2. Phosphogypsum Conversion to P-CSH

A total of 15 g of phosphogypsum was weighed out and added to 150 mL of deionized water; the mixture was then ultrasonicated for 5 min to promote dispersion. Using a Ca/Si molar ratio of 1.0, sodium metasilicate nonahydrate was accurately weighed and 150 mL of solution was prepared. The phosphogypsum suspension was swiftly mixed into the sodium metasilicate solution, stirring continuously for 30 min. This mixture was transferred to a 500 mL sealed hydrothermal reactor and the reaction was maintained at 90 °C for 6 h. Upon completion of the reaction, heating was immediately ceased. The reaction mixture was separated using vacuum filtration, collecting both the crude sodium sulfate liquid and the filter cake. The filter cake was thoroughly washed three times with deionized water. The washed filter cake was dried in a vacuum oven at 60 °C for 24 h and the final product was labeled as P-CSH.

### 3.3. Batch Adsorption Experiment

Due to the notable difference in the adsorption capacities of P-CSH for Pb(II) and Cd(II), the initial concentrations were set at 800 mg/L for Pb(II) and 200 mg/L for Cd(II). Laboratory-prepared deionized water was utilized to create simulated wastewater containing Pb(II) and Cd(II), respectively, set against a background solution of 0.01 mol/L NaNO_3_. The initial pH levels of the solutions were adjusted to a range of 2.0 to 6.0 using 0.1 mol/L solutions of NaOH and HCl. The adsorbent addition was consistently maintained at 0.8 g/L. This study primarily investigated the influence of the initial pH on the removal efficiency of Pb(II) and Cd(II), with the related calculations provided in Equation (16).

(16) $$R(\%)=\frac{{(C}_{0}-C_{e})}{C_{0}}\times 100\%$$

where R (%) represents the removal efficiency; C_0_ (mg/L) represents the initial concentration; and C_e_ (mg/L) represents the equilibrium concentration.

For the adsorption kinetics experiments, 0.16 g of P-CSH was accurately weighed and separately introduced into 200 mL of simulated wastewater solutions containing either Pb(II) or Cd(II). Samples were taken from the mixture at various time intervals—5, 10, 30, 60, 120, 240, 480, 720, 960, 1200, and 1440 min—and approximately 10.0 mL of each suspension was filtered using a 0.22 μm syringe filter. The filtrate’s concentrations of Pb(II) or Cd(II) were determined by flame atomic absorption spectrophotometry (AAS, TAS-986, PERSEE, China). The adsorption capacities of P-CSH for Pb(II) and/or Cd(II) at the different time points were calculated using Equation (17). Each experimental iteration was conducted in triplicate, and the average results were recorded.

(17) $$Q_{t}=\frac{{(C}_{0}-C_{t})\times V}{m}$$

where Q_t_ (mg/g) is the adsorption capacity at time t; C_0_ (mg/L) is the initial concentration; C_t_ (mg/L) is the residual concentration at time t; m (g) is the mass of the adsorbent; and V (L) is the volume of the solution.

For the adsorption isotherm experiments, simulated wastewater samples featuring various initial concentrations of Pb(II) (ranging from 600 to 1000 mg/L at increments of 50 mg/L) and Cd(II) (ranging from 100 to 500 mg/L at increments of 50 mg/L) were prepared, with an adjusted initial pH of 5.0. A measured amount of 0.024 g of P-CSH was added to each 30 mL sample of these prepared waters containing different concentrations of Pb(II) and Cd(II). The mixtures were oscillated for a period of 1440 min, after which the concentrations of residual Pb(II) and Cd(II) in the wastewater were evaluated. Each experiment was conducted in triplicate, and the results were averaged to ensure reliability and accuracy of the data. The adsorption capacity of P-CSH for Pb(II) or Cd(II) was then calculated using Equation (18).

(18) $$Q_{e}=\frac{{(C}_{0}-C_{e})\times V}{m}$$

where Q_e_ (mg/g) is the equilibrium adsorption capacity; C_0_ (mg/L) is the initial concentration; C_e_ (mg/L) is the equilibrium concentration; m (g) is the mass of the adsorbent; and V (L) is the volume of the solution.

For competitive or synergistic adsorption experiments, we prepared composite simulated wastewater with variable initial concentrations of Pb(II) and Cd(II). Specifically, the concentration of Cd(II) was consistently set at 200 mg/L, while that of Pb(II) varied across nine levels: 600, 650, 700, 750, 800, 850, 900, 950, and 1000 mg/L. Conversely, when the concentration of Pb(II) was constant at 800 mg/L, the concentration of Cd(II) alternated between 100 and 500 mg/L, in increments of 50 mg/L. The initial pH of these wastewater samples was adjusted to 5.0. For each experimental setup, 0.024 g of P-CSH was added to 30 mL of the varying concentration wastewater solutions. These mixtures were then oscillated for 1440 min. Subsequently, the concentrations of remaining Pb(II) and Cd(II) were analyzed. Each experiment was conducted in triplicate, and the results were averaged to ensure reliability and accuracy of the data.

To examine the influence of coexisting ions on adsorption efficacy, the fixed parameters included an adsorbent dosage of 0.8 g/L, an initial pH of 5.0, and starting concentrations of 800 mg/L for Pb(II) or 200 mg/L for Cd(II). The reactions were conducted at 30 °C and oscillated continuously for 1440 min. Variations were made solely in the types (Na^+^, K^+^, Ca^2+^, and Mg^2+^) and concentrations (0, 1, 10, and 100 mmol/L) of background ions to assess their impact on the adsorption behaviors of Pb(II) and Cd(II).

### 3.4. Stepwise Desorption and Recovery of Pb(II) and Cd(II)

To prepare 5.0 L of synthetic wastewater consisting of 800 mg/L of Pb(II) and 200 mg/L of Cd(II), the initial pH was adjusted to 5.0 and the background ion concentration was set at 0.01 mol/L NaNO_3_. A total of 4.0 g of P-CSH was combined with this synthetic wastewater, and the mixture was maintained at 30 °C for a 24 h reaction period. Subsequently, the adsorption product (P-CSH/Pb+Cd) was filtered, washed, and dried, and it was utilized for the stepwise desorption and recovery of Pb(II) and Cd(II).

First, the P-CSH/Pb+Cd was placed in a 1.0 mol/L NaOH solution at 80 °C and stirred continuously for 2 h to maintain the reaction. Immediately following the reaction, the mixture was filtered to separate the Pb(II) desorption alkaline solution from the alkaline leaching residue. The Pb(II) desorption solution was allowed to stand at room temperature for 6 h, then the resulting precipitate was filtered, washed, and dried. After washing and drying the alkaline leaching residue, it was transferred into a 0.02 mol/L EDTA solution to recover Cd(II) and eliminate residual calcium. The recovery efficiencies (%) for Pb(II) and Cd(II) were subsequently calculated using Equation (19).

(19) $$\eta =\frac{C_{2}V_{2}}{\omega_{1}m_{1}}\times 100$$

where η (%) represents the recovery efficiency of Pb(II) or Cd(II); $\omega_{1}$ (wt.%) represents the content of Pb(II) or Cd(II) in the solid sample; $m_{1}$ (g) refers to the mass of the solid sample; $C_{2}$ (g/L) indicates the concentration of Pb(II) or Cd(II) in the solution; and $V_{2}$ (L) is the volume of the leachate.

### 3.5. Characterization

The phase composition of the solid samples was characterized through X-ray Diffraction Spectroscopy (XRD, Ultima IV, Rigaku, Tokyo, Japan), within a scan range of 5–60° at a scanning speed of 0.05°/s. The microstructure and elemental distribution of the samples were examined using Scanning Electron Microscopy (SEM, ZEISS Gemini 300, Oberkochen, Germany) and Energy Dispersive X-ray Spectroscopy (EDS, OXFORD Xplore, Oxfordshire, UK). The specific surface area and pore size distribution of both phosphogypsum and P-CSH were assessed utilizing a surface area and porosimetry analyzer (BET, ASAP2460, micromeritics, Atlanta, GA, USA). Calculation of the specific surface area employed the Brunauer–Emmett–Teller method across an adsorption data range of P/P_0_ = 0.01–0.95, with the pore size distribution derived from the desorption branch of a nitrogen adsorption isotherm through the Barret–Joyner–Halenda (BJH) method. Changes in the surface functional groups of the solid samples were analyzed using Fourier-Transform Infrared Spectroscopy (FT-IR, Nicolet 670, Madison, WI, USA) within a wavenumber range of 400–4000 cm^−1^. Finally, high-resolution Solid-State Nuclear Magnetic Resonance (MAS NMR, AVANCE II 400M, Bruker, Switzerland) was used to explore the ^29^Si MAS NMR spectra of P-CSH before and after adsorption, utilizing a 4 mm probe and a spinning rate of 10 kHz.

## 4. Conclusions

In this investigation, we successfully transformed phosphogypsum into P-CSH using a straightforward hydrothermal process and employed it to remove and recover Pb(II) and Cd(II) from wastewater. P-CSH demonstrated substantial adsorption capacity for Pb(II) and Cd(II), achieving maximum adsorption capacities of 989.3 mg/g and 290.3 mg/g, respectively. The adsorption kinetics for both heavy metals followed a pseudo-second-order model, indicating that the process is predominantly controlled by chemical reactions. Additionally, the adsorption isotherm adhered to the Langmuir model, indicating that the adsorption occurred through monolayer adsorption. Notably, since P-CSH utilizes identical adsorption sites for Pb(II) and Cd(II), their simultaneous adsorption results in competitive interactions. P-CSH also exhibited remarkable resistance to interference from cations commonly found in wastewater, such as Na^+^, K^+^, Ca^2+^, and Mg^2+^, which did not significantly impact the adsorption of Pb(II) and Cd(II). The primary mechanisms underpinning the adsorption of Pb(II) and Cd(II) onto P-CSH were identified as chemical precipitation and surface complexation, with no involvement of electrostatic adsorption. During the adsorption process, the C-S-H of P-CSH reactively engages with H^+^ and H_2_O in the solution, releasing Ca^2+^ and OH^−^. This interaction triggers the transformation of the bridging Q_p_^2^ tetrahedral structure within C-S-H to a Q^4^ structure, thus maintaining the adsorbent’s porous structure and facilitating ongoing release of Ca^2+^ and OH^−^. Moreover, the adsorbed products were effectively separated and recovered from wastewater using a stepwise desorption process. The residue post-recovery, primarily consisting of quartz and amorphous SiO_2_, could be dissolved through pressurized alkali leaching and can subsequently be reused in the synthesis of P-CSH.

## Figures and Tables

**Table 1:** Adsorption kinetic parameters for adsorption of Pb(II) and Cd(II) by P-CSH.

Model	Parameter	Pb(II)	Cd(II)
Pseudo-first-order	k_1_ (min^−1^)	0.003413	0.003052
	Q_e_ (mg/g)	448.2	168.6
	R^2^	0.9715	0.9763
Pseudo-second-order	k_2_ (g/(mg·min))	3.27 × 10^−5^	6.24 × 10^−5^
	Q_e_ (mg/g)	954.2	244.1
	R^2^	0.9996	0.9972
Intra-particle diffusion	C_1_ (mg/g)	162.4	17.9
	k_i1_ (mg/(g·min^0.5^))	52.0802	11.8370
	R^2^	0.9397	0.9846
	C_2_ (mg/g)	532.9	86.9
	k_i2_ (mg/(g·min^0.5^))	16.2893	5.3534
	R^2^	0.9678	0.9834
	C_3_ (mg/g)	815.6	156.8
	k_i3_ (mg/(g·min^0.5^))	3.3091	2.1892
	R^2^	0.9310	0.9676

**Table 2:** Adsorption isotherms parameters for adsorption of Pb(II) and Cd(II) by P-CSH.

Model	Parameter	Pb(II)	Cd(II)
Langmuir	Q_m_ (mg/g)	993.1	291.5
	K_L_ (L/mg)	0.6735	0.5097
	R^2^	0.9995	0.9994
Freundlich	K_F_(mg^(1−1/n)^·L^1/n^·g^−1^)	808.3	172.2
	n	26.14	9.54
	R^2^	0.9776	0.9494
Temkin	K_T_ (L/mg)	3.60 × 10^10^	4366.8
	b	75.57	117.33
	R^2^	0.9734	0.9829

## Data Availability

The original contributions presented in the study are included in the article; further inquiries can be directed to the corresponding authors.
